# *In silico *analysis highlights the frequency and diversity of type 1 lantibiotic gene clusters in genome sequenced bacteria

**DOI:** 10.1186/1471-2164-11-679

**Published:** 2010-11-30

**Authors:** Alan J Marsh, Orla O'Sullivan, R Paul Ross, Paul D Cotter, Colin Hill

**Affiliations:** 1 Teagasc, Moorepark Food Research Centre, Fermoy, Cork, Ireland; 2 Microbiology Department, University College Cork, Cork, Ireland; 3 Alimentary Pharmabiotic Centre, Cork, Ireland

## Abstract

**Background:**

Lantibiotics are lanthionine-containing, post-translationally modified antimicrobial peptides. These peptides have significant, but largely untapped, potential as preservatives and chemotherapeutic agents. Type 1 lantibiotics are those in which lanthionine residues are introduced into the structural peptide (LanA) through the activity of separate lanthionine dehydratase (LanB) and lanthionine synthetase (LanC) enzymes. Here we take advantage of the conserved nature of LanC enzymes to devise an *in silico *approach to identify potential lantibiotic-encoding gene clusters in genome sequenced bacteria.

**Results:**

In total 49 novel type 1 lantibiotic clusters were identified which unexpectedly were associated with species, genera and even phyla of bacteria which have not previously been associated with lantibiotic production.

**Conclusions:**

Multiple type 1 lantibiotic gene clusters were identified at a frequency that suggests that these antimicrobials are much more widespread than previously thought. These clusters represent a rich repository which can yield a large number of valuable novel antimicrobials and biosynthetic enzymes.

## Background

Bacteriocins are bacterially produced peptide antibiotics. Two major classes of gram-positive bacteriocins have been recognised, Class I undergo significant post-translationally modifications while the Class II are unmodified [1,2]. The majority of the class I bacteriocins are lantibiotics; small peptides containing internal bridges resulting from the formation of (β-methyl)lanthionine residues. The structural gene encodes a ribosomally synthesised precursor prepeptide which is generically named LanA. This prepeptide contains a leader sequence at the N-terminus, which is ultimately cleaved, and a propeptide at the C-terminus. Many or all of the serine and threonine residues within the propeptide are dehydrated to form dehydroalanine (Dha) and dehydrobutyrine (Dhb), respectively. When these modified residues interact with an intrapeptide cysteine, a thioether bond is formed resulting in the formation of lanthionine (Lan, from Dha) or β-methyl lanthionine (meLan, from Dhb).

The lantibiotics and lantipeptides (lanthionine containing peptides which lack antimicrobial activity) can be divided into four groups according to the nature of the enzymes which catalyse (me)Lan formation [3]. In the case of type 1 lantibiotics two enzymes are involved; LanB, the lanthionine dehydratase which catalyses the dehydration of the amino acids, and LanC, the lanthionine synthetase which catalyses thioether formation. Type 2 lantibiotics contain a single LanM enzyme which performs both functions. Type 3 and 4 are lantipeptides which are also catalysed by distinct enzymes such as the RamC-like and LanL enzymes [4,5]. The type 1 and 2 lantibiotics can also be further subdivided on the basis of the amino acid sequence of the unmodified propeptide. In the case of the type 1 lantibiotics, five such subgroups have been identified, each of which is named after the corresponding prototypical lantibiotic; the nisin-like, epidermin-like, Pep5-like, streptin-like and planosporocin-like lantibiotic subgroups [2,6]. The nisin-like group is named for nisin A, which is the most extensively studied bacteriocin and is currently sold in more than 50 countries as a food preservative [7]. In addition to LanA, B and C, other proteins involved in the the production of nisin A and other type 1 lantibiotics include LanP, a serine protease that cleaves the leader from the propeptide; LanT, an ABC transporter responsible for the transport of the lantibiotic precursor across the cell membrane; LanIEFG encode proteins involved in immunity and; LanK, a histidine kinase and LanR, a response regulator, that together operate as a two-component regulatory system. LanD enzymes, such as that responsible for the oxidative decarboxylation of C-terminal cysteines in epidermin [8], are less common.

Given the broad antimicrobial spectrum of many lantibiotics, the possibility of applying lantibiotics in a medicinal capacity has become the subject of much attention. This is supported by an enhanced understanding of their mechanisms of action [9] and the dearth of novel antibiotics. Of the type 1 lantibiotics, nisin, mutacin and planosporicin have been shown to be active against multi-drug resistant gram-positive pathogens [10-12], Pep5 and epidermin inhibit *Staphylococcus epidermidis *adhesion to catheters [13] and epidermin and gallidermin are active against *Propionibacterium acnes *[14]. Other lantibiotics, or their producer strains, have been used as food preservatives and as oral and gastrointestinal antimicrobials/probiotics [15-17]. As a consequence of this increased interest in lantibiotics, a concerted effort has taken place to identify new and improved forms of these peptides. Culture based screening strategies have in the past been responsible for the identification of lantibiotics produced by bacteria isolated from diverse microbial niches including the oral cavity, intestine, soil, kefir grains and milk [12,18-21]. Recently, an alternative means of identifying novel lantibiotics has emerged as a consequence of the increasing generation and availability of genomic and metagenomic sequence data. The availability of such information has recently led to the identification of the type 1 epidermin-like lantibiotic, Bsa [22] as well as type 2 lantibiotics such as haloduracin [23,24], licheniciden [25,26], as well as a range of cyanobacteria-associated lantipeptides [27]. This has prompted the development of on-line tools and repositories such as BAGEL and BACTIBASE to facilitate such screening strategies [28-31]. Notably, although an *in-silico *screen for *lanM *genes has recently resulted in the identification of 61 novel type 2 lantibiotic-like gene clusters [25], a corresponding screen for type 1 lantibiotics has not yet been described. Here we address this issue by screening for clusters containing genes homologous to the nisin A biosynthetic genes *nisB *(representing *lanB*) and *nisC *(representing *lanC*). In each case, the regions flanking the newly identified *lanB*/*lanC *genes were subjected to further *in silico *analysis to determine if they are potential lantibiotic/lantipeptide-associated gene clusters. This included a search of nearby open reading frames (orfs) which might encode a corresponding LanA, defined as being of relatively short length (approx 60 amino acids) and containing an uneven distribution of cysteine, threonine and serine amino acids within the propeptide region. Using this approach, 27 novel type 1 lantibiotic/lantipeptide-encoding clusters were identified. Subsequent screening using the newly identified LanA, B and C homologs as driver sequences revealed a further 22 gene clusters, resulting in a total of 49 putative novel type 1 lantibiotic clusters. Significantly, many of these clusters are present in species, genera and phyla not previously associated with lantibiotic/lantipeptide production and are predicted to encode peptides which represent completely new type 1 subclasses.

## Results and Discussion

### *In silico *screen for *lanC *genes

An *in silico *screen for LanC homologues, using the NisC sequence as a driver, resulted in the identification of 56 homologues. Of these 7 have previously been associated with lantibiotic production, 11 were orphan homologs (in that no other lantibiotic-associated genes were identified in close association) (Table 1), 9 were encoded within a cluster in which no *lanA *could be detected (Table 2) and one cluster contained two LanCs (but no structural peptide). The remaining 27 potential lantibiotic/lantipeptide-encoding gene clusters all contained putative *lanA*, *B *and *C *genes (Table 3). The genes flanking the 27 novel *lanC*-like genes were subjected to further bioinformatic analysis to determine the presence of other orfs that share homology with genes linked to lantibiotic production or immunity. While these 27 clusters are the primary focus of this *in silico *analysis, the sequences of the newly identified LanA, B and C proteins associated with these clusters were in turn used for further *in silico *screens. This approach uncovered an additional 22 clusters (Table 4) that were also predicted to be novel lantibiotic/lantipeptide-encoding clusters, thereby yielding a total of 49 novel type 1 clusters.

**Table 1:** A selection of bacterial genomes in which isolated genes encoding LanA, LanB or LanC homologs were identified

Clusterless Homologs	Accession No.	LanA only	LanB only	LanC only
*Anoxybacillus flavithermus *WK1	NC_011567		Aflv_2440	
*Bacillus *sp. B14905	NZ_AAXV00000000		BB14905_21668	
*Paenibacillus larvae *subsp. *larvae *BRL-230010	NZ_AARF00000000			Plarl_010100024193
*Lactobacillus crispatus *MV-1A-US	NZ_ACOG00000000			HMPREF0507_00422
*Lactobacillus crispatus *JV-V01	NZ_ACKR00000000			HMPREF0506_0642
*Haliangium ochraceum *DSM 14365	NC_013440			Hoch_3102
*Haliangium ochraceum *DSM 14365	NC_013440			Hoch_4144
*Haliangium ochraceum *DSM 14365	NC_013440		Hoch_0066	
*Pedobacter *sp. BAL39	NZ_ABCM00000000		PBAL39_02527	
*Peptoniphilus lacrimalis *315-B	NZ_ADDO00000000		HMPREF0628_0526	
*Peptoniphilus lacrimalis *315-B	NZ_ADDO00000000		HMPREF0628_0527	
*Lactococcus lactis *subsp. *lactis *KF147	NC_013656			
*Frankia alni *ACN14a	NC_008278		FRAAL2701	
*Frankia *sp. CcI3	NC_007777			Francci3_0205
*Frankia *sp. CcI3	NC_007777			Francci3_3997
*Peptoniphilus lacrimalis *315-B	NZ_ADDO00000000		HMPREF0628_0527	
*Bifidobacterium longum *subsp. *infantis *ATCC 15697	NC_011593			
*Streptomyces *sp. AA4	NZ_ACEV00000000			
*Streptococcus pneumoniae *CGSP14	NC_010582			
*Saccharopolyspora erythraea *NRRL 2338	NZ_ABFV00000000			SACE_4959
*Kordia algicida *OT-1	NZ_ABIB00000000			KAOT1_07113
*Streptococcus dysgalactiae *GGS_124	NC_012891			SDEG_0295
*Microscilla marina *ATCC 23134	NZ_AAWS00000000		M23134_07394	
*Microscilla marina *ATCC 23134	NZ_AAWS00000000		M23134_05752	
*Microscilla marina *ATCC 23134	NZ_AAWS00000000		M23134_07275	
*Microscilla marina *ATCC 23134	NZ_AAWS00000000		M23134_01545	
*Microscilla marina *ATCC 23134	NZ_AAWS00000000			M23134_07404
*Streptomyces *sp. Mg1	NZ_ABJF00000000		SSAG_05771	
*Frankia *sp. EuI1c	NZ_ADDL00000000	FraEuI1cDRAFT_6351		
*Geobacillus *sp. Y412MC52	NZ_ACNM00000000	GYMC52DRAFT_3129		
*Geobacillus *sp. Y412MC61	NC_013411	GYMC61_1158		
*Streptococcus pyogenes *M1 GAS	NC_002737	SPy_1083		
*Streptomyces griseus *subsp. *griseus *NBRC 13350	NC_010572			SGR_6574
*Clostridium kluyveri *DSM 555	NC_009706			CKL_3505
*Spirosoma linguale *DSM 74	NC_013730		Slin_0903	
*Spirosoma linguale *DSM 74	NC_013730		Slin_2131	

**Table 2:** Gene clusters encoding LanB and LanC, but not LanA, homologs

Species (Cluster No.)	Accession No.	LanB	LanC
*Frankia alni *ACN14a I	NC_008278	FRAAL2701	FRAAL2700
*Frankia *sp. CcI3 IV	NC_007777	Francci3_2033	Francci3_2032
*Frankia *EAN1pec II	NC_009921	Franean1_2799	Franean1_2800
*Frankia *sp. EuI1c II	NZ_ADDL00000000	FraEuI1cDRAFT_6786	FraEuI1cDRAFT_6785
*Bacillus clausii *KSM-K16	NC_006582	ABC3559	ABC3558
*Clostridium cellulovorans *743B	NZ_ACPD00000000	ClocelDRAFT_0447	ClocelDRAFT_0445/_0452
*Bacillus cereus *AH1273	NZ_ACMT00000000	bcere0030_58380	bcere0030_58400
*Bacillus thuringiensis serovar berliner *ATCC 10792	NZ_ACNF00000000	bthur0008_53920	bthur0008_53930
*Bacillus thuringiensis *IBL 200	NZ_ACNK00000000	bthur0013_59170	bthur0013_59180
*Streptococcus pyogenes *MGAS9429	NC_008021	MGAS9429_Spy0926	MGAS9429_Spy0924
*Catenulispora acidiphila *DSM 44928	NC_013131	Mentioned; Caci_4205	Caci_4204
*Frankia *sp Cc13 V	NC_007777	Francci3_3530	Francci3_3531
*Microscilla marina *ATCC 23134	NZ_AAWS00000000	M23134_05752	M23134_05756
*Staphylococcus capitis *SK14	NZ_ACFR00000000	STACA0001_2327	STACA0001_2326
*Streptomyces *sp. Mg1 I	NZ_ABJF00000000	SSAG_03540	SSAG_03541

**Table 3:** Bacterial genomes in which 27 uncharacterised type 1 lantibiotic clusters were identified following a NisC-led in silico screen

Species (Cluster No.)	Accession No.
*Frankia *alni ACN14a (II)	NC_008278
*Frankia *sp Cc13 (I)	NC_007777
*Frankia *sp Cc13 (II)	NC_007777
*Frankia *sp Cc13 (III)	NC_007777
*Frankia *EAN1pec (I)	NC_009921
*Frankia *EAN1pec (III)	NC_009921
*Frankia *sp. EuI1c (I)	NZ_ADDL00000000
*Salinispora arenicola *CNS-205*	NC_009953
*Stackebrandtia nassauensis *DSM 44728 (I)	NC_013947
*Stackebrandtia nassauensis *DSM 44728 (II)	NC_013947
*Streptomyces clavuligerus *ATCC 27064 (I)	NZ_ADGD00000000
*Streptomyces clavuligerus *ATCC 27064 (II)	NZ_ADGD00000000
*Streptomyces coelicolor *A3(2) (I)	NC_003888
*Streptomyces coelicolor *A3(2) (II)	NC_003888
*Streptomyces *sp. Mg1 (II)	NZ_ABJF00000000
*Streptomyces griseoflavus *Tu4000 (I)	NZ_ACFA00000000
*Streptomyces griseoflavus *Tu4000 (II)	NZ_ACFA00000000
*Streptomyces griseoflavus *Tu4000 (III)	NZ_ACFA00000000
*Bacillus cereus *F65185	NZ_ACMO00000000
*Bacillus mycoides *DSM 2048	NZ_ACMU00000000
*Clostridium perfringens *CPE str. F4969	NZ_ABDX00000000
*Enterococcus faecalis *Fly1	NZ_ACAR00000000
*Geobacillus kaustophilus *HTA426	NC_006510
*Geobacillus thermodenitrificans *NG80-2	NC_009328
*Geobacillus *sp. G11MC16	NZ_ABVH00000000
*Streptococcus thermophilus *LMG 18311*	NC_006448
*Chitinophaga pinensis *DSM 2588 I	NC_013132

*The existence of a lantibiotic gene cluster within these strains has been referred to briefly, [36] and [79,80] respectively, but these clusters have not been the focus of a detailed bioinformatic analysis.

**Table 4:** Bacterial genomes in which 22 additional type 1 lantibiotic gene clusters were identified following an in silico screen using the LanA, B, and C homologs, corresponding to the clusters referred to in Table 3, as leader sequences

Species (Cluster No.)	Accession No.
*Thermomonospora curvata *DSM 43183	NC_013510
*Frankia *EAN1pec (IV)	NC_009921
*Streptomyces viridochromogenes *DSM 40736	NZ_ACEZ00000000
*Streptomyces *sp. SPB74	NZ_ABJG00000000
*Streptomyces lividans *TK24	NZ_ACEY00000000
*Catenulispora acidiphila *DSM 44928	NC_013131
*Streptomyces *sp. Mg1 (III)	NZ_ABJF00000000
*Nocardiopsis dassonvillei *subsp. *dassonvillei *DSM 43111	NZ_ABUI00000000
*Micromonospora aurantiaca *ATCC 27029 (I)	NZ_ADBZ00000000
*Micromonospora aurantiaca *ATCC 27029 (II)	NZ_ADBZ00000000
*Bacillus cereus *AH1272	NZ_ACMS00000000
*Staphylococcus aureus *subsp. *aureus *D139	NZ_ACSR00000000
*Staphylococcus aureus *subsp. *aureus *H19	NZ_ACSS00000000
*Actinomyces *sp. oral taxon 848	NZ_ACUY00000000
*Parachlamydia acanthamoebae *str. Hall's coccus	NZ_ACZE00000000
*Corynebacterium lipophiloflavum *DSM 44291	NZ_ACHJ00000000
*Staphylococcus aureus *A9765	NZ_ACSN00000000
*Chitinophaga pinensis *DSM 2588 (II)	NC_013132
*Spirosoma linguale *DSM 74	NC_013730
*Pedobacter heparinus *DSM 2366	NC_013061
*Kordia algicida *OT-1	NZ_ABIB00000000
*Microscilla marina *ATCC 23134	NZ_AAWS00000000

All except one of the 27 gene clusters revealed following the initial screen were located within the genomes of *Firmicutes *and *Actinobacteria*. The exception was *Chitinophaga pinensis *DSM 2588 of the phylum *Bacteroidetes*. Of the other 26, the genera most commonly associated with lantibiotic production were *Bacillus*, *Geobacillus*, *Clostridium*, *Enterococcus*, *Streptococcus*, *Frankia *and *Streptomyces*. In many cases the novel clusters associated with a specific genus, such as those found on the *Streptomyces *and *Frankia *genomes, showed at least some similarity to each other. It was also noted that several of the genomes in which a cluster was located also contained an additional cluster(s) (Table 3), or other genes predicted to encode additional LanA, B or C proteins (Table 2), elsewhere in the genome. The 27 clusters are described below and are grouped according to the phylum and genus of the associated strain.

### Type 1 lantibiotic gene clusters in *Actinobacteria*

#### Identification of novel *Frankia*-associated lantibiotic gene clusters

**Figure 1:**
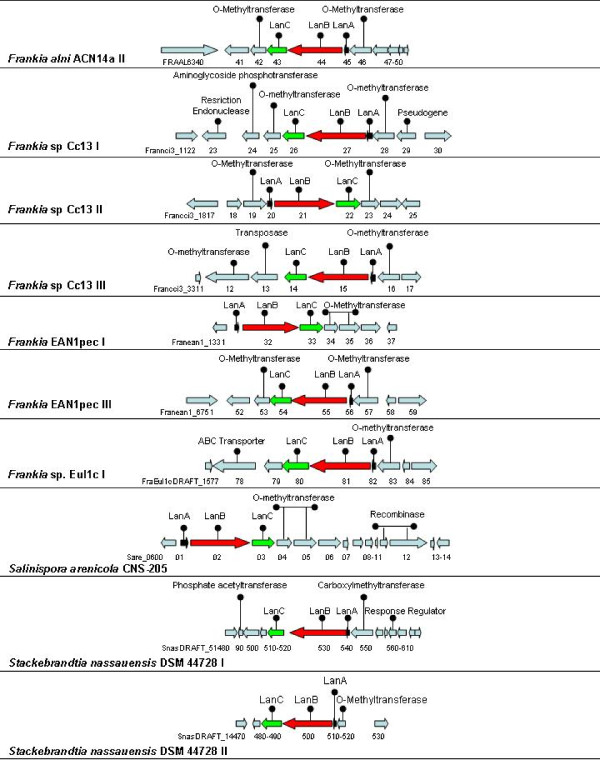
**Diagramatic representation of the non-streptomyces *Actinobacteria *type 1 lantibiotic operons, found in the original NisC screen, which contain genes predicted to encode a structrural peptide LanA, and the modification enzymes LanB and LanC**.

The *Frankia *are nitrogen-fixing, root nodule-forming filamentous *Actinobacteria *that live in symbiosis with actinorhizal plants. All species of *Frankia *are closely related [32]. To date, four *Frankia *genomes have been sequenced, i.e. *Frankia alni *ACN14a, *Frankia *sp. EAN1pec, *Frankia *sp. Cc13 and *Frankia *sp. EUI1c, and although no *Frankia*-associated bacteriocins have previously been reported, a number of predicted lantibiotic clusters can be found in each case (Figure 1) in addition to a number of apparently LanB- and LanC-encoding genes which do not have an accompanying *lanA *(Table 2). This latter phenomenon could be a result of the frequent rearrangements which occur in *Frankia *strains [32]. Of the clusters identified, many resemble clusters associated with another genus of *Actinobacteria*, the *Streptomyces*.

*Frankia alni *ACN14a: The genome sequence of *F. alni *ACN14a [32] contains one complete cluster, *F. alni *ACN14a II which includes the predicted LanA prepropeptide, FRAAL6345, FRAAL6344 and FRAAL6343 (encoding a putative LanB and LanC, respectively). The LanA peptide does not resemble any of the previously characterised type 1 lantibiotic propeptides but is 79% identical to Franean1_0057 of *Frankia *sp. EAN1pec (see below). The LanB and LanC proteins resemble those associated with other *Frankia*, as well as *Streptomyces*, clusters. The LanC protein is also 62% identical to Tcur_4648 of *Thermomonospora curvata *DSM 43183 (NC_013510), which itself appears to be encoded by an orf within a novel lantibiotic gene cluster. The ACN14a II cluster is also predicted to encode two proteins which share homology with O-methyltransferases (FRAAL6342 and FRAAL6346). O-methyltransferases contribute to the production of a number of non-ribosomal antibiotics [33,34] and catalyse the methylation of hydroxyl group(s) on deoxysugar rings to protect the reactive hydroxyl group from undesired modifications and can alter the solubility and pharmacokinetic properties of the resulting molecule [35]. Although O-methyltransferases have not previously been associated with lantibiotic production, this study reveals that many *Actinobacteria*-associated type 1 clusters possess genes predicted to encode these enzymes.

*Frankia *sp. Cc13: The *Frankia *sp. Cc13 genome [32] contains three gene clusters of interest (*Frankia *sp. Cc13 I, *Frankia *sp. Cc13 II and *Frankia *sp. Cc13 III). Two O-methyltransferase-encoding genes were identified in each case and it was noted that the associated LanB and LanC proteins are similar to one another and to numerous others predicted to be produced by *Frankia *and *Streptomyces *species. In contrast, there is a lack of homology between the three *lanA *genes. The *lanA *gene from cluster I was not previously annotated and was only identified following closer inspection of the DNA sequence. The cluster II-associated LanA, Francci3_1820, most closely resembles *Frankia *sp. EuI1c FraEuI1cDRAFT_6351 (69% identity) while the third, and also previously unannotated LanA appears to be one of an extended group of *Frankia*- and *Streptomyces*-associated LanAs that includes *Frankia *sp. EuI1c FraEuI1cDRAFT_1582 (56% identity).

*Frankia *EAN1pec: The *Frankia *EAN1pec genome [32] contains 4 putative LanB-encoding genes three of which correspond to potential lantibiotic/lantipeptide-associated gene clusters (*Frankia *EAN1pec I, *Frankia *EAN1pec III and *Frankia *EAN1pec IV) which again resemble those of *Streptomyces *and other *Frankia *species, and contain O-methyltransferase-encoding genes. Within the first cluster, a putative LanA prepropeptide, encoded by a previously unannotated orf located between Franean1_1331 and the LanB determinant, is homologous to a number of other LanAs, including Sare_0601 of *Salinispora arenicola *CNS-205 (55% identity). The cluster is also noteworthy be virtue of the presence of two LanC-encoding genes, Franean1_1333 and Franean1_1336. Within *Frankia *EAN1pec III, the LanA peptide, encoded by Franean1_6756 is 42% identical to FraEuI1cDRAFT_6351 of *Frankia *sp. EuI1c while finally, a screen using *F. alni *ACN14a FRAAL6345 as a driver led to the identification of yet another cluster (consisting of at least Franean1_0057-0055) which closely resembles cluster II of *F. alni *ACN14a II.

*Frankia sp. *EuI1c: *Frankia *sp. EuI1c contains a single putative lantibiotic/lantipeptide gene cluster (*Frankia *sp. EuI1c I) which again contains LanB, C and O-methyltransferase genes typical of *Frankia *and *Streptomyces *clusters. The associated LanA homolog (FraEuI1cDRAFT_1582) is notable by virtue of being 46% identical to SSCG_03316, a known LanA of *Streptomyces clavuligerus *ATCC 27064 while a gene encoding an ABC transporter related protein (FraEuI1cDRAFT_1578) is also present.

#### Identification of novel *Salinispora *-associated lantibiotic gene clusters

*Salinispora *are marine *Actinobacteria*. There are two recognised species, *S. tropicalis *and *S. arenicola. *Representatives have been sequenced in each case and genes predicted to encode non-lantibiotic bacteriocins have been identified in both cases [36]. The existence of a putative lantibiotic/lantipeptide cluster, between Sare_0602 and Sare_0623, in the genome of *S. arenicola *CNS-205 was noted previously [36]. However, this cluster has not been the subject of a detailed bioinformatic characterisation. Our analysis reveals that Sare_0601 apparently encodes a LanA peptide which is 88% identical to that encoded by MicauDRAFT_5818 of *Micromonospora aurantiaca *ATCC 27029. The proteins encoded by Sare_0602 (LanB) and Sare_0603 (LanC) also resemble other ATCC 27029-associated proteins (encoded by MicauDRAFT_5819 (71% identity) and MicauDRAFT_5820 (75% identity)), thereby revealing an additional novel cluster in *Micromonospora*, a genus better known for its production of non-ribosomal antibiotics such gentamycin and netamycin [37] (Table 4).

#### Identification of novel *Stackebrandtia*-associated lantibiotic gene clusters

*Stackebrandtia *are aerobic, non-motile *Actinobacteria *which have been isolated from soil [38]. There are only 2 associated species i.e. *S. albiflava *and *S. nassauensis *and *in silico *analysis of *S. nassauensis *DSM 44728 (NC_013947) reveals the presence of two similar clusters (*S. nassauensis *DSM 44728 I and *S. nassauensis *DSM 44728 II) (Figure 1). The hypothetical LanA, encoded by Snas_5416, of the first cluster showed a singular homology of 78% identity to Snas_3601 of the second cluster. The corresponding LanBs (Snas_5417 and Snas_3602) are 62% identical while the LanCs (Snas_5418 and Snas_3603) are 68% identical.

#### Identification of novel *Streptomyces*-associated lantibiotic gene clusters

**Figure 2:**
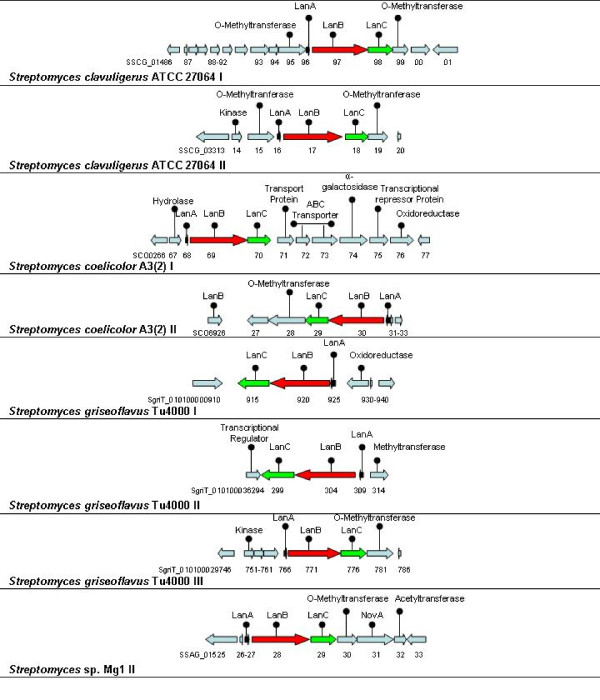
**Diagramatic representation of the *Streptomyces *type 1 lantibiotic operons, found in the original NisC screen, which contain genes predicted to encode a structrural peptide LanA, and the modification enzymes LanB and LanC**.

Bacteria from the genus *Streptomyces*, comprising over 500 species, are filamentous, high G-C bacteria found frequently in soil and rotting vegetation. They are the most numerous and ubiquitous soil bacteria [39]. *Streptomyces *are also responsible for the production of over two-thirds of the clinically useful antibiotics of natural origin (e.g., neomycin, chloramphenicol) [40]. Although a number of *Streptomyces*-associated bacteriocins, such as ancovenin [41] and cinnamycin [42], have been identified, this number is relatively small considering the size of the genus. As was apparent above, our *in silico *analysis has revealed that many *Streptomyces *possess potentially lantibiotic-encoding gene clusters which resemble those found in *Frankia*. Once again, the majority of these clusters contain O-methyltransferases (Figure 2).

##### *Streptomyces clavuligerus *ATCC 27064

*S. clavuligerus *is an aerobic, mesophillic *Streptomyces *sp. While there have been no previous reports of bacteriocin production by this species, two lantibiotic clusters were found to be present on the genome of *S. clavuligerus *ATCC 27064. In the first of these clusters, the associated hypothetical LanA, B and C proteins (SSCG_01498-01496) are 63%, 42% and 50% identical to the corresponding proteins of *Frankia *sp. CcI3 II. BLAST analysis of these proteins also revealed another novel cluster in *Streptomyces viridochromogenes *DSM 40736 corresponding to SvirD4_23440 (LanA; 50% identity), SvirD4_23449 (LanB; 36% identity) and SvirD4_23454 (LanB; 45% identity) (Table 4). The second *S. clavuligerus *cluster, which contains SSCG_03316 (LanA), SSCG_03317 (LanB) and SSCG_03318 (LanC), resembled clusters present in a number of other strains such as that of *Streptomyces griseus *subsp. *griseus *NBRC 13350 [43] (73%, 56% and 64% identity, respectively). BLAST analysis of these sequences also led to the identification of yet another novel cluster in *Streptomyces *sp. SPB74 (SSBG_01041 [LanA] 69% identity and SSBG_01042 [LanB] 58% identity).

##### *Streptomyces coelicolor *A3(2)

*Streptomyces coelicolor *A3(2) (NC_003888) is the best characterised representive of its genus [44] and was the first *Streptomyces *strain to have its genome sequenced [45]. Although bacteriocins/bacteriocin-like peptides are known to be produced by this species (e.g. the class III morphogenic peptide SapB [4]), such peptides have not previously been associated with this strain. Here BLAST analysis revealed the presence of two lantibiotic/lantipeptide clusters (*S. coelicolor *A3(2) I and *S. coelicolor *A3(2) II). The first of these clusters, containing SCO0268 (LanA), SCO0269 (LanB) and SCO0270 (LanC), very closely resembles *Streptomyces griseoflavus *Tu4000 cluster II (see below). Subsequent BLAST searches with the A3(2) cluster I-associated proteins led in turn to the discovery of an almost identical cluster in *Streptomyces lividans *TK24 which contains SSPG_07329 (LanA; 100% identity), SSPG_07328 (LanB; 99% identity) and SSPG_07327 (LanC; 100% identity) (Table 4). The second cluster, *Streptomyces coelicolor *A3(2) II, is predicted to encode two LanA peptides, (SCO6932 [43aa] and SCO6931 [59aa]) which are 97% identical to each other, as well as LanB (SCO6930) and LanC (SCO6929) proteins with homology (39-46% identity) with corresponding proteins associated with *Frankia *sp. CcI3. Such analysis also revealed another cluster of interest in the actinomycete, *Catenulispora acidiphila *DSM 44928 (NC_013131; [Table 4]).

##### *Streptomyces griseoflavus *Tu4000

Three lantibiotic/lantipeptide clusters were identified on the genome of this anaerobic, terrestrial *Streptomyces*. Although the LanA encoded within the first cluster (SgriT_010100000925) does not significantly resemble any other protein, the associated LanB (SgriT_010100000920) and LanC (SgriT_010100000915) proteins are homologous to the corresponding proteins of *S. coelicolor *A3(2) cluster I and Tu4000 cluster II. BLAST searches using the cluster I proteins as drivers also resulted in the identification of several additional clusters in *Nocardiopsis dassonvillei *DSM 43111 (NdasDRAFT_3161 [LanB] 30% identity), *Streptomyces sp. *Mg1 (SSAG_05771 [LanB] 37% identity) and two clusters on the genome of *M. aurantiaca *ATCC 27029 (NZ_ADBZ00000000; MicauDRAFT_5820 and MicauDRAFT_3008 [both LanBs] 35% identity). In addition to the components of the second cluster referred to above, an associated LanA (SgriT_010100036309) was also noted. In addition to the Tu4000 I and A3(2) I clusters, this cluster is also highly identical to that of *S. lividans *TK24 (SSPG_07329 [LanA] 97% identity; SSPG_07328 [LanB] 87% identity and SSPG_07327 [LanC] 89% identity). The LanA associated with the final cluster (SgriT_010100029766) again bears no homology with any other known peptides whereas the LanB (SgriT_010100029771) and LanC (SgriT_010100029776) corresponded to those of *Frankia *sp. EAN1pec II (39% and 44% identity, respectively).

### Type 1 lantibiotic gene clusters in *Firmicutes*

#### Identification of novel *Bacillus*-associated lantibiotic gene clusters

**Figure 3:**
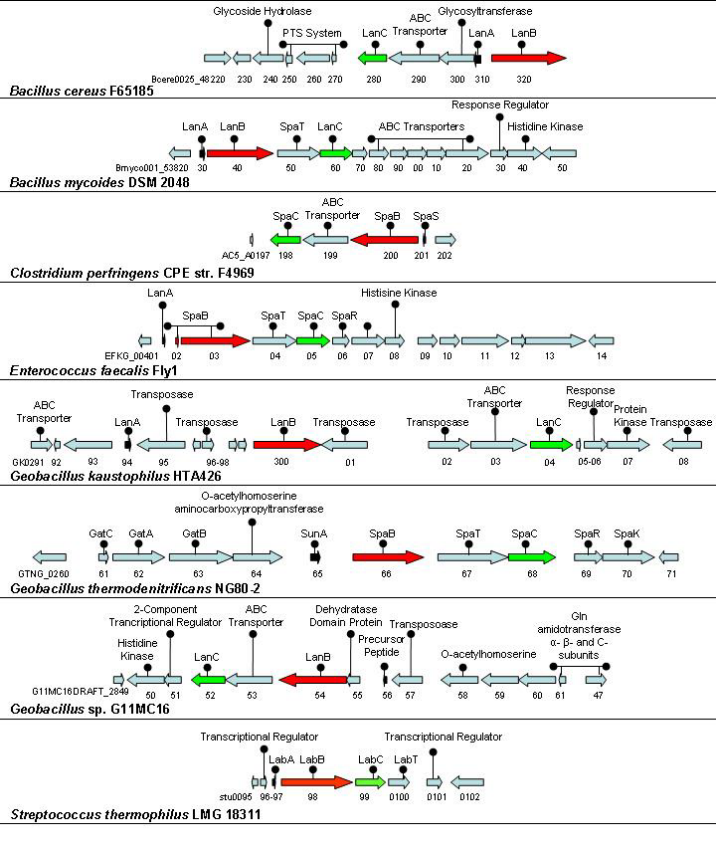
**Diagramatic representation of the *Firmicutes *type 1 lantibiotic operons, found in the original NisC screen, which contain genes predicted to encode a structrural peptide LanA, and the modification enzymes LanB and LanC**.

*Bacillus *is a large and diverse genus of rod-shaped, sporulating, obligate aerobes which contains both free living and pathogenic species. A number of type 1 lantibiotics have previously been characterized in this genus (e.g. subtilin [46] and ericin [47]). The NisC-driven screen highlighted the presence of a type 1 lantibiotic cluster in the genomes of two *Bacillus *strains i.e. *Bacillus cereus *F65185 and *Bacillus mycoides *DSM 2048 (Figure 3). Bioinformatic analysis of these clusters revealed two further clusters in *B. cereus *ATCC 14579 and *B. cereus *AH1272.

##### *Bacillus cereus *F65185

*B. cereus *F65185 is a mesophilic bacterium sourced from a human wound containing one lantibiotic/lantipeptide cluster which is unusual in that 3 orfs separate the putative LanB and C genes and the two have a divergent orientation. The predicted LanA (bcere0025_48310) does not resemble any other known lantibiotic prepropeptides. The LanB homolog (bcere0025_48320) resembles a putative LanB associated with *Clostridium cellulovorans *743B (ClocelDRAFT_0452, 30% identity) while the predicted LanC (bcere0025_48280) most closely resembles two further 743B proteins (ClocelDRAFT_0452, 34% identity and ClocelDRAFT_0446, 28% identity). However the 743B strain lacks an associated LanA. Further BLAST analysis with the F65185-associated LanB highlighted the presence of a related protein within thiocillin-encoding gene cluster in *B. cereus *ATCC 14579 [48].

##### *Bacillus mycoides *DSM 2048

*B. mycoides *is a non-motile, non-pathogenic, saprophytic *Bacillus*, strains of which have been investigated with a view to their application as biological pesticides. Although representatives of this species have been associated with bacteriocin production [49], there are no published reports of lantibiotic-producing *B. mycoides*. The DSM 2048 genome contains a lantibiotic/lantipeptide cluster that contains putative *lanA*, *B*, *C *and other lantibiotic-associated genes which is very similar to other novel clusters in *B. cereus *AH1272 and *B. cereus *AH1273. Bmyco0001_53830 is the predicted prepropeptide and is 100% identical to the products of the *B. cereus *AH1272-associated bcere0029_28240 and bcere0029_28250. It is also 58% identical to prepropeptides associated with several *Staphylococcus*-associated Bsa lantibiotics, such as BsaA2_RF122 _of *S. aureus *RF122 [22]. It is thus apparent that the LanA is a member of the epidermin-like peptides. In addition to homologues in *B. cereus *AH1273 and AH1272, use of the DSM 2048-associated LanB and LanC sequences as drivers also surprisingly highlighted a gene cluster present in *Actinomyces *sp. oral taxon 848 (Table 4). In addition to homology with respect to LanB and LanC proteins (HMPREF0972_00932 and HMPREF0972_00933, respectively), the proposed LanA (HMPREF0972_00931; 86aa) is 43% identical to bmyco0001_53830 and bcere0029_28240 and bcere0029_28250 of *Bacillus mycoides *DSM 2048 and *Bacillus cereus *AH1272, respectively.

#### Identification of novel *Clostridium*-associated lantibiotic gene clusters

The *Clostridia *are gram postitive anaerobic, endospore-forming *Firmicutes *of which there are approximately 100 species. These include important pathogens such as *Clostridium difficile, Clostridium perfingens *and *Clostridium tetani*. Several *Clostridium*-associated bacteriocins have been characterised from this genus [50-52], but no type 1 lantibiotic producers have been identified to date. Here, bioinformatic analysis revealed one *Clostridium*-associated lantibiotic/lantipeptide cluster, located on the genome of *C. perfringens *CPE str. F4969 (Figure 3).

##### *C. perfringens *CPE str. F4969

*C. perfringens *is one of the leading causes of food-borne illness in the developed world, usually as a result of the improper sterilization of canned foods in which endospores have germinated. It can also be responsible for wound and surgical infections [53,54]. The predicted LanA (AC5_A0201) of strain F4969 is most closely related to the LanAs of *B. mycoides *DSM 2048 and *B. cereus *AH1272 (51% identity) while the proteins encoded by AC5_A0200 (LanB) and AC5_A0198 (LanC) most closely resemble proteins associated with *Geobacillus *sp. G11MC16 (G11MC16DRAFT_2954 and G11MC16DRAFT_2952; 34% and 35% identity respectively). Surprisingly, BLAST analysis also highlighted the presence of a related LanB homolog encoded within a lantibiotic/lantipeptide-like gene cluster in the genome of *Parachlamydia acanthamoebae *(phlylum *Chlamydiae*) str. Hall's coccus (pah_c028o031; 25% identity). Adjacent genes of note within the *P. acanthamoebae *cluster include pah_c028o029 (LanA) and pah_c028o030 (LanC).

#### Identification of novel *Enterococcus*-associated lantibiotic gene clusters

The enterococci are gram positive lactic acid bacteria which are common commensal organisms in the intestines of humans but can also be pathogens. Many *Enterococcus*-associated bacteriocins (enterocins) have been identified [55]. Only one *Enterococcus*-associated lantibiotic, the type 2 peptide cytolysin, has been identified to date [56]. Here we describe genes which potentially encode the first type 1 *Enterococcus*-associated lantibiotic (Figure 3).

##### *Enterococcus faecalis *Fly1: *E. faecalis*

Fly1 (NZ_ACAR00000000) is a non-motile, facultative anaerobe. Within its genome we identified a previously unannotated LanA determinant, through analysis of raw sequence data. The corresponding peptide is homologous to *C. perfringens *CPE str. F4969 (AC5_A0201; 68% identity), as well as a number of epidermin-like LanAs in other bacilli. The putative LanB protein is split across two orfs, EFKG_00402 (80 amino acids) and EFKG_0403 (942 amino acids), with both components most closely resembling the N-terminus of the dehydratase of *Streptococcus pyogenes *MGAS10270, MGAS10270_Spy0922. It is unclear whether the apparent frameshift in the Fly1 *lanB *is genuine or the result of a sequencing error. The LanC-like EFKG_00405, was most closely related to the corresponding protein in *G. thermodenitrificans *NG80-2 (SpaC GTNG_0268; 35% identity).

#### Identification of novel *Geobacillus*-associated lantibiotic gene clusters

Geobacilli are thermophillic (45-70°C), aerobic, spore-forming *Firmicutes*. They have been isolated from various terrestrial and marine environments, in geothermal, temperate and permanently cold habitats. Reclassified in 2001 [57], these bacteria are of industrial interest as sources of thermostable enzymes. Bacteriocins have been identified in *Geobacillus stearothermophilus *[58] and *Geobacillus thermoleovorans *[59], and while screening for LanM-producing gene clusters has highlighted the potential existence of a number of type 2 lantibiotics [25], associated type 1 lantibiotics have not previously been described. Here, three putative type 1 lantibiotic/lantipeptide-encoding clusters within the genomes of *Geobacillus kaustophilus *HTA426, *Geobacillus thermodenitrificans *NG80-2 and *Geobacillus sp. *G11MC16 (Figure 3) are described.

##### *Geobacillus kaustophilus *HTA426

*G. kaustophilus *grows optimally in aquatic environments at 60°C with an upper temperature limit of 74°C. From a lantibiotic persepective, genome xsequencing of HTA426 revealed a hypothetical protein annotated as a 'lantibiotic precursor' GK0294. Our analysis revealed that this putative LanA is 91% identical to another prepropeptide encoded by the closely located GK0286 gene. It is also 100% identical to orphan 'lantibiotic precursor' homologs (GYMC52DRAFT_3129 and GYMC61_1158) in *Geobacillus *sp. Y412MC52 and *Geobacillus *sp. Y412MC61, respectively. More distantly related LanAs (79% identity) are also associated with the genomes of *Geobacillus thermodenitrificans *NG80-2 (GTNG_0265) and *Geobacillus *sp. G11MC16 (G11MC16DRAFT_2956). The homology between the *Geobacillus *LanAs is highest within the leader regions, but, as is the case with nisin-, epidermin- and streptin-like lantibiotics, a conserved serine and CTPGC motif in the N-terminus of the propeptide is present, which is believed to be involved in the binding of these lantibiotics to lipid II in the cell wall in gram positive bacteria [60]. BLAST analysis of the GK0286-encoded LanA highlighted the presence of another potential lantibiotic/lantipeptide cluster in *Corynebacterium lipophiloflavum *DSM 44291 (57% identity with HMPREF0298_1795). Within the HTA426 cluster, the proteins predicted to be encoded by GK0300/301 (an apparently frameshifted *lanB*) and GK0304 are homologous to those associated with many other geobacilli. It was also noted that this cluster is less condensed than typical lantibiotic gene clusters in that there are insertions of 7, 5 and 3 genes (predicted to encode many transposases and small, hypothetical proteins) between the lantibiotic associated genes.

##### *Geobacillus thermodenitrificans *NG80-2 and *Geobacillus sp. G11MC16:*

*G. thermodenitrificans *are facultative soil bacteria with denitrification qualities. Representatives of this species grow between 45°C and 73°C (optimum 65°C). NG80-2 was isolated from a deep-subsurface oil reservoir in Dagang oilfield, Northern China [61] and on the basis of *in-silico *analysis is potentially the producer of both a type 1 (see below) and type 2 lantibiotic [25]. Our analysis reveals that the type I lantibiotic/lantipeptide operons in *G. thermodenitrificans *NG80-2 and *Geobacillus sp. *G11MC16 are very highly conserved. The two LanAs are 100% identical and the homology between these, and indeed the associated B and Cs, and the corresponding *G. kaustophilus *HTA426 proteins is discussed above. It was noted that the *lanB *of *Geobacillus sp. G11MC16 *is apparently frameshifted (G11MC16DRAFT_2955 (176aa) and G11MC16DRAFT_2954 (848aa)) but that this is not the case in *G. thermodenitrificans *NG80-2 (GTNG_0266).

#### Identification of novel *Staphylococcus*-associated lantibiotic gene clusters

The staphylococci are non-sporeforming, non-motile *Firmicutes*. The genus *Staphylococcus *contains 33 species, most of which are harmless and reside normally on the skin and mucous membranes of humans and other organisms. However, staphylococci can also cause a wide variety of diseases either through toxin production or penetration and are a common cause of food poisoning and nosocomial infections. Several strains of *Staphylococcus epidermidis *have been shown to be producers of type 1 lantibiotics, including epidermin [62], Pep5 [63], epicidin 280 [64] and epilancin K7 [65], gallidermin was isolated from *S. gallinarum *[14] while Staphylococcin Au26 [66] and Bsa [22] were isolated from *S. aureus*. BLAST analysis has revealed that several other *S. aureus *strains possess gene clusters similar to those associated with Bsa and Bsa_RF122 _[22]. These clusters were identified in *S. aureus *A9765, D139 and H19. In A9765, SAPG_01762 and SAPG_01760 correspond to the BsaA1 and BsaA2 peptides of *S. aureus *MW2 (97% and 100% identity, respectively). The precursor peptides of the D139 (SATG_00575 and SATG_00574; 76% identical to each other) and H19 (SAUG_01228 and SAUG_01229; 76% identical to each other) strains are 100% identical. The peptides encoded by SATG_00575 and SAUG_01229 are 93% identical to BsaA1_RF122 _of *S. aureus *RF122 (93% identity) while those corresponding to.SATG_00574 and SAUG_01228 are 100% identical to BsaA2_RF122_.

#### Identification of novel *Streptococcus*-associated lantibiotic gene clusters

These facultative anaerobes of the phylum *Firmicutes *are spherical in shape and grow in long chains. Many species are part of the normal commensal flora of the mouth, skin, intestine and upper respiratory tract of humans but the genus also includes numerous human pathogens such as *Streptococcus pneumoniae*, *pyogenes *and *agalactiae*. The streptococci are known to producers of type 1 lantibiotics [67,68], such as streptin [69], some mutacins [21,70-72], nisin U and nisin U2 [73], as well as several non-lantibiotic bacteriocins. Here we discuss two clusters, identified in strains of *S. pyogenes *and *S. thermophilus *LMG 18311.

##### *Streptococcus pyogenes *MGAS10270

*S. pyogenes *(or Group A *Streptococcus*, GAS) is the cause of many important human diseases ranging from mild superficial skin infections to life-threatening systemic diseases. Bacteriocin production by these strains may give them a competitive advantage against the natural skin microbiota. It has previously been established that many *S. pyogenes *strains, as well as strains of *Streptococcus salivarius*, produce the type 2 lantibiotic salivaricin A or closely related variants [16]. The type 1 streptins (1 and 2) and type 2 streptococcin A-FF22 are also *S. pyogenes *associated [69,74]. Here our analysis focuses on a type 1 cluster within the genome of *S. pyogenes *MGAS10270 [75]. This includes MGAS10270_Spy0919, which is 100% identical to the propeptide sequence of streptin. While this lantibiotic is thus not novel, subsequent BLAST searches were revealing in that they highlighted the presence of a LanA with 97% identity in *S. pyogenes *MGAS10750 (MGAS10750_Spy0955) which is contained within a cluster which also encodes a LanB (MGAS10750_Spy0958) and LanC (MGAS10750_Spy0957).

##### *Streptococcus thermophilus *LMG 18311

*S. thermophilus *is a thermophillic, non-pathogenic *Streptococcus*. It is of major importance to the fermented dairy food industry. A number of non-lantibiotic bacteriocins (thermophilins) from this species have been characterized, including thermophilin 347 [76], thermophilin A [77] and thermophilin ST-1 [78]. Strain LMG 18311 was sequenced in 2004 and at the time it was noted that bacteriocin production was one of the characteristics that distinguishes it from strain CNRZ1066 [79]. While the existence of a putative lantibiotic/lantipeptide gene cluster in LMG 18311 has been reported [79,80], this cluster (Figure 3) has not been the focus of a detailed *in silico *analysis. The associated LanA, encoded by stu0097, is homologous with that predicted to be encoded by SPCG_0144 of *S. pneumoniae *CGSP14 (88% identical) which, on the basis of previous *in silico *analysis, is also within a lantibiotic gene cluster [81]. The LanB protein (Stu0098) is 73% identical to SPCG_0145 of *S. pneumoniae *CGSP14 and 97% identical to a truncated LanB associated with *S. thermophilus *CNRZ1066 [79].

### Type 1 lantibiotic gene clusters in *Bacteroidetes*

The Bacteroidetes are a highly diverse phylum found in soil, seawater and the skin and intestines of animals. The Bacteroidales class, which includes the genus *Bacteroides*, are the best-studied of the phylum. Bacteroides comprises the most substantial portion of the human gastrointestinal tract [82] some of which are opportunistic pathogens [83].

#### Identification of novel *Chitinophaga*-associated lantibiotic gene clusters

**Figure 4:**
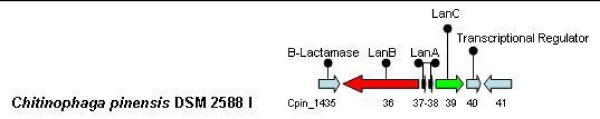
**Diagramatic representation of the *Chitinophaga pinensis *(*Bacteroidetes*) type 1 lantibiotic operons, found in the original NisC screen, which contain genes predicted to encode a structrural peptide LanA, and the modification enzymes LanB and LanC**.

*Chitinophaga *are rod-shaped mesophiles of the phylum *Bacteroidetes *which are are noted for their ability to degrade chitin [84]. There have been no reports to date of bacteriocin production by any of the 10 *Chitinophaga *species. *Chitinophaga pinensis *DSM 2588 (NC_013132) is unusual in that it appears to be a *Bacteroidetes *possessing genes encoding a type 1 lantibiotic (Figure 4). There are two predicted LanA peptides, corresponding to Cpin_1438 and Cpin_1437, which are 50% identical as a consequence of similar N-terminii. Adjacent orfs of note include Cpin_1435 and Cpin_1440, predicted to encode a β-lactamase and a 2-component transcriptional regulator of the LuxR family, respectively. BLAST analysis of the associated LanB and LanC proteins (Cpin_1436 and Cpin_1439 respectively) revealed another putative LanB (Cpin_3392; 36% identity) and LanC (Cpin_3397; 23% identity) encoded within the same genome. Within this second *C. pinensis*-associated cluster, Cpin_3393 possess a number of features which suggest that it may be a LanA-encoding gene. Interestingly, BLAST analysis of the Cpin_3397-encoded LanC also led to the identification of a number of additional homologs apparently encoded within the genomes of strains not previously associated with lantibiotic production. The genome of another *Bacteroidetes*, *Spirosoma linguale *DSM 74, is notable in that it contains 4 putative LanB-encoding genes and 5 putative LanC-encoding genes. Of these only one LanB protein (Slin_4704; 31% identity) and one LanC protein (Slin_4705; 26% identity), are encoded within what appears to be a novel lantibiotic-associated gene cluster. This cluster contains 3 potentially LanA-encoding genes, Slin_4706-4708. Slin_4706 and Slin_4707, which are identical and share 58% identity with Slin_4708. The genome of another *Bacteroidetes *species, *Pedobacter heparinus *DSM 2366 (NC_013061), contains a cluster encoding two LanBs (Phep_0556 and Phep_0557; 37% and 36% identity, respectively), a LanC (Phep_0555; 33% identity) and a potential LanA (Phep_0553; no significant BLAST hits). A cluster within *Kordia algicida *OT-1 contains LanB (KAOT1_15523; 36% identity), LanC (KAOT1_15518; 30% identity) and LanA determinants (KAOT1_15533; no significant BLAST hits) while *Microscilla marina *ATCC 23134 potentially has five associated LanB homologs, but of these, the LanB corresponding to M23134_03921 (28% identity) was the only one to be located in close proximity to one of multiple LanC proteins (M23134_03925; 22% identity). The putative LanA, M23134_03926, does not resemble any other known proteins.

### Phylogenetics of LanABC

**Figure 5:**
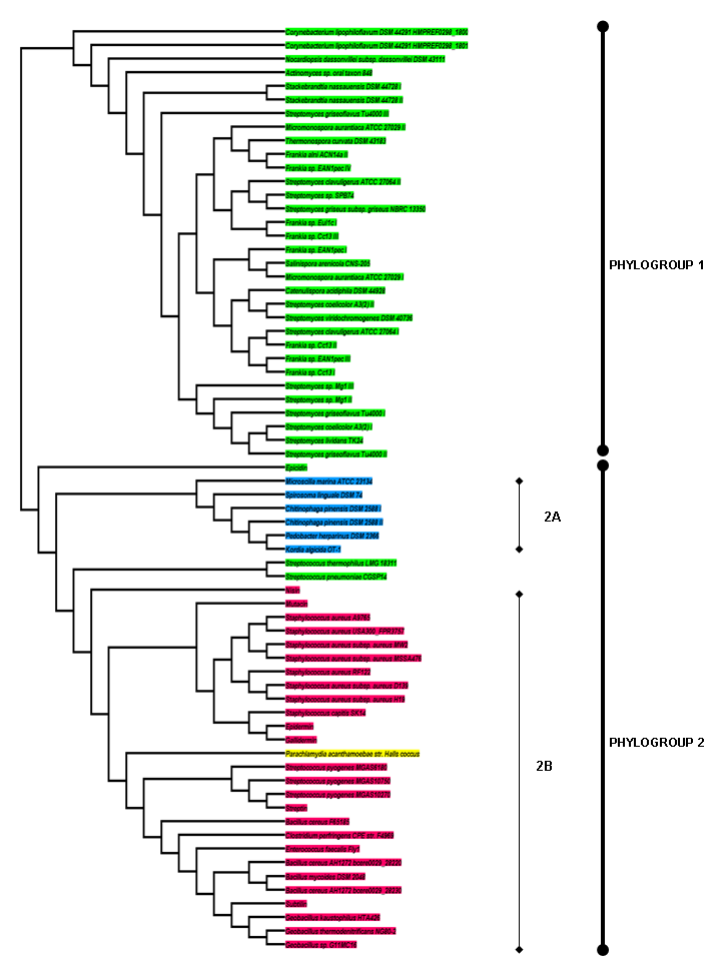
**Cladogram of all the LanB enzymes from clusters encountered during the screen**. Also included are dehydratases from some well-known lantibiotics. Green = *Actinobacteria*; Pink = *Firmicutes*; Blue = *Bacteroidetes*; Yellow = *Chlamydiae*

The conserved nature of LanB and LanC proteins facilitated a phylogenetic analysis of their relatedness. The resultant cladogram of LanB enzymes (all those identified in both screens, as well as a number of LanBs from previously analysed clusters) highlights the existence of two distinct phylogroups (Figure 5). The first phylogroup contains *Actinobacteria*-associated LanBs, all of which are from strains not previously known to be producers of lantibiotics/lantipeptides. The second contains a variety of lanthionine synthetases associated with known lantibiotics (nisin, subtilin, epidermin etc), some uncovered by previous *in silico *analysis (e.g. *S. pneumoniae *CGSP14 [81]), novel clusters from genera with which lantibiotic production has previously been attributed as well as genera not previously associated with lantibiotic production. Within this second phylogroup one finds two subgroups; one consisting of *Bacteroidetes*-associated LanBs (2A) and a second consisting of *Firmicutes*-associated LanB's (2B) as well as that from *P. ancanthamoeba*. Among the *Firmicutes*-associated LanBs further subclustering is evident. One common branch contains three offshoots; (i) *Bacillus*/*Geobacillus*/*Enterococcus*/*Clostridium*, (ii) *S. pyogenes *and (iii) *P. acanthamoebae *str. Hall's coccus LanBs. The *Staphylococcus *LanBs and that of mutacin 1140 (those associated with epidermin-like peptides) also form a distinct subgroup as do those encoded with the genomes of *S. thermophilus *LMG 18311 and *S. pneumoniae *CGSP14. Curiously the epicidin (*S. epidermidis*) LanB does not group with any other LanB.

**Figure 6:**
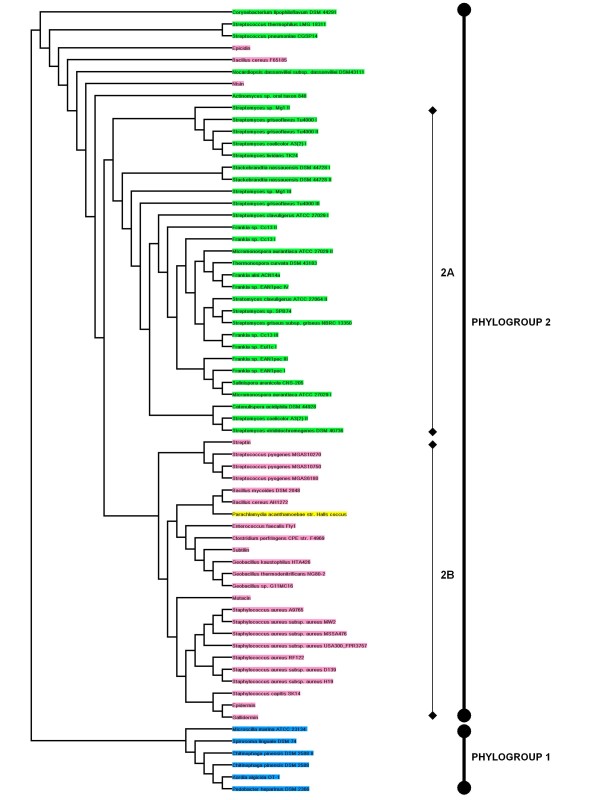
**Cladogram of all the LanC enzymes from clusters encountered during the screen, as well as some from well-known lantibiotics**. Green = *Actinobacteria*; Pink = *Firmicutes*; Blue = *Bacteroidetes*; Yellow = *Chlamydiae*

The cladogram of the corresponding lanthionine synthetases (LanCs) is quite similar to that of the dehydratases (Figure 6). All can be positioned into one of two phylogroups (phylogroups 1 and 2). Phylogroup 1 contains six *Bacteroidetes*-associated LanC's. In contrast phylogroup 2 is large and can be further divided into group 2A, which are *Actinobacteria*-associated, and the *Firmicutes*-associated group 2B. Further subgrouping follows the patterns identified from analysis of the LanB cladogram. 8 exceptional LanCs that avoid subgrouping include those associated with *C. lipophiloflavum *DSM 44291, *S. thermophilus *LMG 18311/*S. pneumoniae *CGSP14, epicidin, *B. cereus *F65185, *N. dassonvillei *subsp. *dassonvillei *DSM 43111, nisin-producing lactococci and *Actinomyces *sp. oral taxon 848.

**Figure 7:**
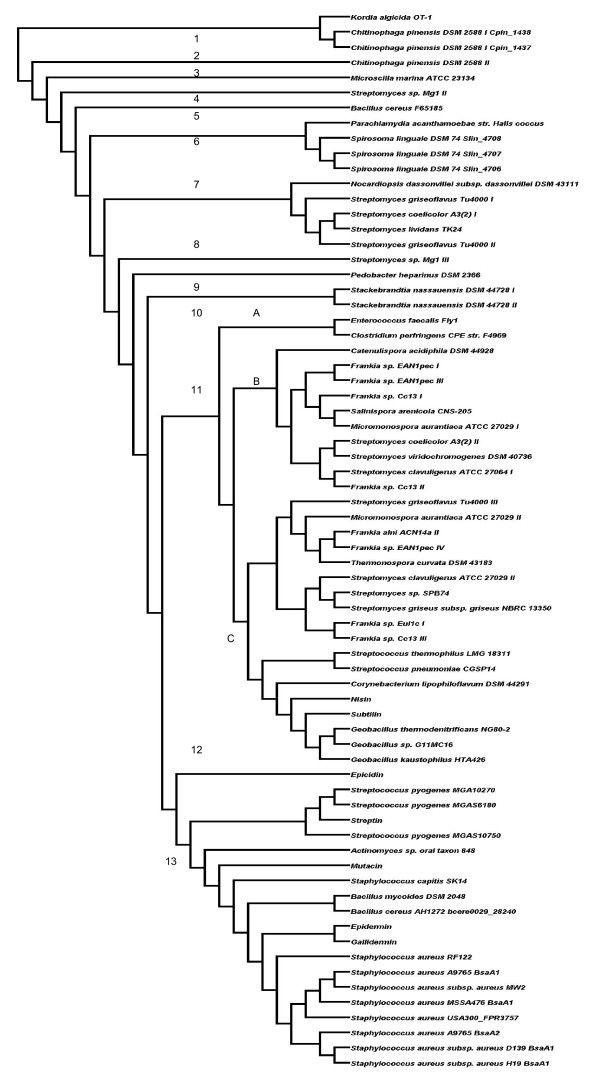
**A cladogram of all the LanA prepeptides identified in this study, as well as a number of previously identified LanAs**.

A cladogram of the less highly conserved LanAs revealed 13 major branches, several of which contain only one corresponding LanA (Figure 7). Notably the various phylogroups do not group in a phylum specific manner to the same extent as was evident in LanB and LanC cladograms. The largest phylogroups, i.e. phylogroups 11 and 13, are those containing the nisin-like and epidermin-like peptides, respectively. While phylogroup 13 is, with the exception of the *Actinomyces *sp. oral taxon 848-associated LanA, composed of *Firmicutes*-associated LanAs, phylogroup 11 contains LanAs from both *Firmicutes *and *Actinobacteria*. This phylogroup contains three subgroups, with subgroup 11A containing LanAs from *E. faecalis *Fly1 and *C. perfringens *F4969, 11B contains *Actinobacteria*-associated LanAs and 11C contains both *Actionobacteria*- and *Firmicutes*-associated LanAs. Of the other phylogroups, phylogroups 6 and 7 are largest and contain *Chlamydiae*/*Bacteroidetes*- and *Actinobacteria*-associated LanAs, respectively.

### Alignment of LanABC

The availability of a significant number of LanA, B and C protein sequences enabled further *in silico *analysis to identify conserved motifs and residues. Alignment of 66 LanB proteins highlighted a number conserved motifs which are summarised in Table 5. A YxxR motif (corresponding to residues 80-83 of NisB) is conserved in 89% of the LanB enzymes, while a GxG motif (363-365) is present in 92% of LanBs, with the LanB of *C. lipophiloflavum *being exceptional by virtue of lacking both glycine residues. A GRF motif (463-465) is fully conserved in 86% of LanBs with the *Streptomyces *sp. Mg1 III LanB being the only protein to lack this motif. An RxTPFG motif (87-94) is present in 77% of LanBs but is completely absent from the LanBs of *Geobacillus *sp. G11MC16, *Streptomyces *sp. Mg1 III, *M. aurantiaca *and *C. lipophiloflavum*. A FxxxYG motif (342-347) is present in 82% of LanBs and, although present in only 50% of LanBs, a PxxxRxxNV (501-509) motif is at least partially conserved in many such proteins i.e. 94% contain the proline, 71% contain the NV residues and the least conserved is the arginine with 71% conservation. Elsewhere, there is a RFL motif (585-587) conserved in 51% of LanBs, a RYG motif (826-828) conserved in 85% of LanBs and a HxxxNR motif (961-966) in 70% of the dehydratases. n addition to these, there are multiple highly conserved residues such as aspartates at residues 121, 299, 648 and 843, prolines at 612 and 639 and a leucine, tryptophan and phenylalanine at 97, 616 and 840.

**Table 5:** Highly conserved residues shared by 66 cluster-associated (including those from the 49 novel clusters referred to in this paper) lanthionine dehydratases (LanB).

From Alignment	NisB Position	% Conservation
**Motifs**		
YxxR	80-83	89%
RxTPFG	87-94	77%
FxxxYG	342-347	82%
GxG	363-365	92%
GRF	463-465	86%
PxxxRxxNV	501-509	50%
RFL	585-587	51%
RYG	826-828	85%
HxxxNR	961-966	70%
**Single Residues**		
R	14	86%
D	121; 299; 648; 843	86%; 94%; 94%; 85%
N	145	86%
L	217	97%
P	612; 639	100%; 95%
E	975	89%
W	616	98%
F	840	95%
V	352	83%

Residues are numbered according to their position in NisB

Alignment of the LanC protein also revealed several conserved regions (summarised in Table 6). Of these, CHG and WCYG motifs were particularly notable. The CHG motif (corresponding to residues 330-333 of NisC) was found to be conserved in 98% of the LanCs. The cysteine^330 ^and histidine^331 ^residues, which act as ligands to the zinc in the active site of NisC, have been shown to be necessary for enzyme activity [85]. The WCYG motif (283-286) was present in 95% of the aligned enzymes. Within the WCYG motif, tryptophan^283 ^(W) and cysteine^284 ^(C) have been shown to be vital to subtilin and nisin biosynthesis (residue numbers refer to location in NisC) [85,86]. It has previously been shown that although alanine subsititution of tyrosine^285 ^(Y) results in enzyme inactivation, a phenylalanine change is tolerated indicating that the presence of an aromatic ring at this position is of key importance [85]. In the same study, a preceding arginine residue (Arg^280^), present in 86% of these enzymes, was found not to be essential for enzyme activity. 92% of LanCs also contained a closely located Gly^289 ^residue. The histidine^212 ^of another highly conserved motif, GxAHGxxG (209-216; conserved in 83% of LanCs), together with a conserved aspartic acid^141 ^(91% of LanCs) are thought to be involved in the electrophilic activation of the carbonyl group of dehydroalanine/dehydrobutyrine or in the protonation of the enolate (thiol substrate) [85]. The HG of this latter motif was conserved in 98% of the enzymes (the exception being *S. aureus *subsp. *aureus *D139). In addition to these, other motifs of note included LxxG (39-42; conserved in 83% of LanCs), GxxxGxxGxxLxL (377-389; 73%) and YDxxxGxxG (140-148; 67%). Highly conserved single residues include Gly^90 ^(94%) and Tryp^258 ^and Tryp^401 ^(83% and 92% respectively).

**Table 6:** Highly conserved residues shared by 66 cluster-associated (including those from 49 novel clusters) lanthionine cyclases. Residues are numbered according to their position in NisC

Conserved Residues	NisC Position	% Conservation
**Motifs**		
LxxG	39-42	83%
YDxxxGxxG	140-148	67%
GxAHGxxG	209-216	83%
WCYG	283-286	95%
CHG	330-332	98%
GxxxGxxGxxLxL	377-389	73%
**Single Residues**		
G	90	94%
W	258; 401	83%; 92%

**Figure 8:**
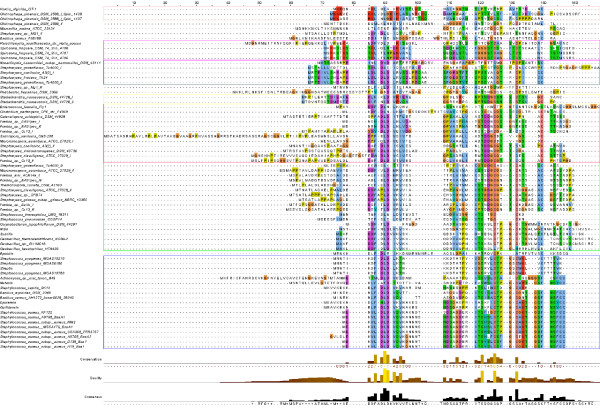
**Alignment of the LanA prepeptides identified in this study, as well as a number of previously identified LanAs**.

Although LanA peptides are less conserved than their modification enzymes, some motifs were evident (Figure 8). A DLD motif present in the leader region of the almost all phylogroup 13 LanAs is also found in many other LanAs. Indeed, the leucine of this motif is conserved across 93% of the 70 aligned peptides. The only peptides lacking this residue are those from *M. marina*, *B. cereus *F65185, *N. dassonvillei *subsp. *dassonvillei *and *P. heparinus *as well as mutacin 1140. The leader regions from many actinobacteria, and especially those from phylogroup 11, are also distinctive as a consequence of the frequent presence of proline residues.

Within the propeptide, the most highly conserved residues are cysteines corresponding to positions 30 and 34 of the Nisin A prepropeptide [60] which significantly are within the lipid II-binding region of the peptide. These cysteines are each found in 86% of the type 1 LanAs. The *Actinobacteria *in phylogroups 10 and 11 also share a conserved or partially conserved DGCG motif in the propeptide region. A less highly conserved AC motif which is closer to the C terminus is also evident. In addition to motifs which are conserved across motifs, a large number of motifs which are conserved within phylogroups are evident.

## Conclusions

The *in silico *strategy adopted here resulted in the initial identification of 56 proteins which share 20-30% identity with NisC. Further investigation of novel LanC proteins in turn led to the identification of even more homologs, revealing novel lantibiotic/lantipeptide associated clusters and establishing the existence of subgroups of LanA, B and C proteins. Theoretically, additional homologs could be identified through a continuation of this method but the rate at which new homologs would be identified would begin to level off. The approach taken led to the identification of 49 novel clusters which, prior to this study, had not been the subject of a detailed bioinformatic analysis. While the *in silico *identification of gene clusters in a strain will not always be confirmed by detection of an associated lantibiotic/lantipeptide, past experience [22-25] suggests that there is likely to be a strong correlation. It is thus anticipated that the peptides produced by these gene clusters will represent a valuable resource, as will be the associated biosynthetic proteins.

This study reveals new details regarding type 1 lantibiotics and their associated clusters. Type 1 lantibiotics have been predominantly associated with the *Firmicutes*, with the *Actinobacteria*-produced planosporicin and microbisporicin being notable exceptions. It is thus interesting to find type 1 clusters distributed among the genomes of bacteria representing four different phyla, the *Actinobacteria*, *Firmicutes*, *Bacteroidetes *and *Chlamydiae*, which have been isolated from a diverse range of habitats including soil, skin, intestines and the deep-sea. Indeed, based on these investigations, it would appear that such clusters are as common among *Actinobacteria *as they are among *Firmicutes*, with *Streptomyces *and *Frankia *sp. being particularly rich sources. The *Actinobacteria *clusters are, in general, quite similar, typically encoding a LanA, B, C and a methyltransferase. The role of the methyltransferase is not clear but may serve to protect specific serine and threonine residues from LanB-mediated dehydration. The presence of five clusters within the genomes of five *Bacteroidetes*, a phylum in which bacteriocin production is purportedly quite rare, is particularly noteworthy. However, the sequencing of additional representatives of this species may well reveal this to be a common feature. The *P. acanthamoebae *cluster is unusual by virtue of its presence in a representative of the *Chlamydiae*. Phylogenetic analysis indicates that the LanB and LanC proteins from this strain are closely related to those of several *Firmicutes *and thus the cluster may originally have been acquired from such a source.

The availability of a much larger collection of LanA, B and C sequences for further *in silico *analysis is also extremely useful for a number of other reasons. In addition to providing greater certainty with respect to the proposed conservation of particular motifs, it also reveals the existence of a greater number of subgroups of sequences than was previously apparent. This is particularly important with respect to LanAs as alignment of these peptides has previously been employed as a means of subgrouping type 1 lantibiotics [2,6]. Ultimately, the most significant outcome has been the number of new type 1 lantibiotic gene clusters. When one considers that less than 25 type 1 lantibiotics had been identified prior to this study, this represents a major expansion. While the genome sequenced strains themselves can be accessed with a view to purifying the associated peptides and/or utilising the biosynthetic machinery, the information gathered will also encourage researchers to include *Actinobacteria *and *Bacteroiodetes *when carrying out wet lab-based screens for novel lantibiotic producers. A combination of this approach and analysis of newly generated bacterial genome sequence data will ensure that many more lantibiotics and lantipetides will soon be discovered which are associated with unusual microorganisms and a wide variety of environments.

## Methods

### Screening of genomic databases

Using the nisin modification enzyme NisC (GenBank accession number CAA79470) as a driver sequence, all fully sequenced genomic sequences (approx. 1178 at time of study; Dec 2009) were mined for homologs using Genomic-BLAST (http://www.ncbi.nlm.nih.gov/sutils/genom_table.cgi). BLASTs were carried out with default parameters; criteria for homolog detection were a threshold of 1e^-7 ^and greater than 20% identity.

### Bioinformatic anlaysis of *lanC*-containing gene clusters

In cases where novel *lanC*-like genes encoding enzymes were identified, the arrangement of adjacent genes was visualised using the genome viewer on NCBI, and individual orfs were subjected to BLAST analysis to identify those potentially involved in lantibiotic production or immunity. The predicted LanA, LanB and LanC proteins from these operons were each in turn used for further *in silico *screens to determine their similarities to corresponding proteins associated with known lantibiotics and to identify additional novel clusters. In instances where a LanC- and LanB-, but not a LanA-, encoding gene were annotated, intergenic regions were inspected following translation by the Seqbuilder program of the DNASTAR Lasergene 8 software package to investigate the presence of potentially unidentified *lanA *genes. The tblastn program was then used to search all sequenced DNA for related peptides.

### Phylogenetic analysis

Protein alignments were generated by MUSCLE [87]. Sequence alignment were viewed and edited for publication with Jalview alignment editor [88]. These alignments were used to establish phylogenetic trees in Phylip [89] which were subsequently visualised using the Dendroscope package [90].

## Authors' contributions

PDC conceived the study and designed the project. OOS participated in the design and coordination of the project. AJM performed the screening of the databases and the bioinformatic and phylogenetic analysis. AJM and PDC wrote the manuscript. OOS, PRR and CH contributed in the preparation of the manuscript. All authors have read and approved the final manuscript.
